# Editorial: Advances in flow diverter interventions for complex intracranial aneurysms: techniques, clinical applications, and outcomes

**DOI:** 10.3389/fneur.2026.1888925

**Published:** 2026-08-27

**Authors:** Ming Wang, Shu Wan

**Affiliations:** 1 Brain Center, Zhejiang Hospital, Hangzhou, China; 2 Zhejiang Province Engineering Research Center for Precision Medicine in Cerebrovascular Diseases, Hangzhou, China

**Keywords:** antiplatelet therapy, complication prevention, flow diverter, intracranial aneurysm, stent-assisted therapy

Flow diverters (FDs) have revolutionized the endovascular management of intracranial aneurysms, particularly complex lesions characterized by atypical morphology, wide necks, blister-like features, dissection, fusiform, or small size—pathologies that pose major challenges to conventional coil embolization and microsurgical clipping. In recent years, technical improvements, device iteration, optimized perioperative monitoring and updated antiplatelet regimens have continuously broadened the indications for FD implantation and improved procedural safety. This Research Topic addresses core issues surrounding FD therapy for complex intracranial aneurysms, covering treatment efficacy, safety profiles, complication prophylaxis, intraoperative evaluation, antiplatelet management, and real-world clinical outcomes across diverse subtypes of complex aneurysms.

A retrospective study by Han et al. evaluated the performance of the pipeline embolization device (PED) in treating ruptured complex intracranial aneurysms. Complex aneurysms were defined as aneurysms that were difficult to treat with conventional endovascular and surgical approaches, including blister-like aneurysms, dissecting aneurysms, fusiform aneurysms, large wide-neck saccular aneurysms (maximum diameter ≥ 10 mm), and small wide-neck saccular aneurysms (diameter ≤ 5 mm) in this study. The application of PED for ruptured complex intracranial aneurysms remains controversial owing to concerns regarding thromboembolic and hemorrhagic risks, with a procedural complication rate of 13.0% and a 2.2% mortality rate attributable to aneurysm rupture. At 90 days, 95.3% of patients achieved favorable clinical outcomes, and a 95.0% complete occlusion rate was documented at a mean follow-up of 7.5 months (Han et al.). These results suggest that PED is a viable treatment option for ruptured aneurysms refractory to conventional therapies, though large-scale comparative clinical trials are still required for further validation.

A systematic review by Maimaitiaili et al. included 10 retrospective analyses. Literature was retrieved from four databases up to July 15, 2025 using combined MeSH and free-text terms. Eligible studies were observational research on ≥20 adults with small intracranial aneurysms ( ≤ 10 mm) receiving FD treatment and reporting relevant outcomes. Large aneurysms, non-target interventions, reviews, case reports and duplicate data were excluded. It was confirmed that FDs achieve a pooled complete/near-complete occlusion rate of 86%, with low rates of treatment-related mortality, hemorrhagic events, ischemic events, stroke, and an overall complication rate of 9%. Favorable functional outcomes were achieved in nearly 98% of patients (Maimaitiaili et al.). Despite substantial heterogeneity across included studies, FDs demonstrate favorable efficacy and safety profiles for the treatment of small intracranial aneurysms.

Stent-assisted embolization for intracranial aneurysms is complicated by thromboembolic events, which are poorly predicted by routine platelet function testing. Berdikhojayev et al. reported a single-center retrospective analysis of 284 adult patients undergoing stent-assisted endovascular embolization, all of whom received preoperative dual antiplatelet therapy (DAPT). Preoperative platelet reactivity was measured via the VerifyNow™ assay: a P2Y12 reaction unit (PRU) < 240 indicated inadequate P2Y12 inhibition, and an aspirin reaction unit (ARU) < 550 suggested suboptimal aspirin efficacy. The novel intraoperative Kazakh ThromboTest (KTT) enables real-time monitoring of thrombotic risk by performing serial angiography at 7 and 15 min after partial stent deployment. In this cohort, the overall stent thrombosis rate was 3.52% (10/284), comprising eight intraoperative and two delayed thrombotic events. The KTT successfully identified all cases of intraoperative stent thrombosis and yielded a high negative predictive value, whereas the VerifyNow™ assay failed to predict such adverse events (Berdikhojayev et al.). As a practical and cost-effective tool, KTT is particularly valuable in medical centers with limited access to standard laboratory testing.

FDs are widely adopted for the treatment of wide-necked intracranial aneurysms. Perioperative DAPT is essential for preventing FD-associated thromboembolic complications; however, prolonged DAPT significantly increases hemorrhagic risk, highlighting the clinical need for safe and timely DAPT discontinuation. An observational study by Fukutome et al. used angioscopy to directly visualize neointimal coverage at 6 months after FD placement and assessed the safety of DAPT discontinuation in eight consecutive patients. The cohort had a mean patient age of 60.5 years and a mean aneurysm diameter of 7.7 mm. Angioscopy verified adequate neointimal coverage (grade ≥1) in all enrolled cases. By contrast, conventional cone-beam computed tomography (CBCT) missed subtle incomplete neointimalization in one patient, owing to its limited resolution for mild neointimal changes. These findings prove that angioscopy is superior to CBCT in detecting thin neointimal layers, and can effectively guide individualized DAPT discontinuation (Fukutome et al.).

Chen et al. analyzed 13 patients with ruptured blood blister-like aneurysms (BBAs) treated with a combined strategy of FD deployment and coil embolization. Most patients achieved favorable mid-term outcomes, with 100% angiographic cure of BBAs and full restoration of arterial wall integrity. Nonetheless, perioperative and postoperative complications occurred in four patients, with an overall incidence of 30.8% (4/13). Among the four patients with preoperative vasospasm, two developed postoperative transient hemiparesis and achieved complete neurological recovery. One patient with preoperative vasospasm developed multiple cerebral infarctions after EVT and died. Another patient who discontinued postoperative antiplatelet therapy suffered multiple infarctions and irreversible severe neurological disability. Overall, ischemic complications are the predominant adverse events after endovascular treatment for ruptured BBAs. Strict adherence to standardized antiplatelet protocols and targeted perioperative management of cerebral vasospasm are therefore critical to improve the safety of EVT for this disease entity (Chen et al.).

A retrospective multicenter analysis by Yao et al. assessed the safety, efficacy, and technical considerations of FDs for proximal middle cerebral artery (M1/M2) non-saccular aneurysms. FD implantation was associated with 100% technical success, a 92.1% complete occlusion rate at a median follow-up of 8.4 months, and 97.7% favorable clinical outcomes at a median follow-up of 25 months, with low perioperative complication rates. This study validates FDs as a viable alternative to surgical intervention for proximal middle cerebral artery (M1/M2) non-saccular aneurysms (Yao et al.).

Juan et al. investigated the Tubridge FD (TFD) for the treatment of unruptured complex intracranial aneurysms. The overall complete occlusion rate was 78.57%, with significantly higher occlusion rates observed in non-saccular than in saccular lesions. Small-to-medium aneurysms (< 10 mm) could be effectively treated with TFD alone. The device exerted minimal impact on branch vessel patency, and 97.01% of patients achieved excellent clinical outcomes (mRS 0–1), confirming the safety and effectiveness of the TFD (Juan et al.).

Collectively, the studies included in this Research Topic demonstrate that FDs achieve high technical success rates and durable angiographic occlusion for ruptured/unruptured, small-sized, blister-like, dissecting, fusiform, and non-saccular aneurysms of the internal carotid and middle cerebral arteries. Perioperative complication and mortality rates are acceptable, and the majority of patients achieve favorable long-term functional outcomes. Notwithstanding these consistent favorable results, most included studies are retrospective in design and exhibit moderate heterogeneity. Larger prospective randomized controlled trials and extended long-term follow-up are necessary to validate these findings, standardize clinical management protocols, and refine patient selection criteria for FD therapy.

In conclusion, modern flow diverter interventions represent a safe, effective, and reliable therapeutic strategy for complex intracranial aneurysms. The evidence synthesized in this Research Topic reinforces the expanding role of flow diversion in neurointerventional practice and facilitates the delivery of personalized patient care.

